# A digital health home intervention for people within the Alzheimer’s disease continuum: results from the Ability-TelerehABILITation pilot randomized controlled trial

**DOI:** 10.1080/07853890.2023.2185672

**Published:** 2023-03-17

**Authors:** Federica Rossetto, Sara Isernia, Olivia Realdon, Francesca Borgnis, Valeria Blasi, Chiara Pagliari, Monia Cabinio, Margherita Alberoni, Fabrizia Mantovani, Mario Clerici, Francesca Baglio

**Affiliations:** a IRCCS Fondazione Don Carlo Gnocchi ONLUS, Milan, Italy; b Department of Human Sciences for Education, Università degli Studi di Milano-Bicocca, Milan, Italy; c Department of Physiopathology and Transplants, Università degli Studi di Milano, Milan, Italy

**Keywords:** Telerehabilitation, Alzheimer’s disease, Mild Cognitive Impairment, Rehabilitation, Disability, Dementia

## Abstract

**Purpose:**

This study tested the efficacy of digital-health home intervention for people within the Alzheimer’s disease (AD)-continuum.

**Methods:**

Thirty people within the AD continuum were randomly assigned to a telerehabilitation (ABILITY; 6 males, M_age_=78.2 ± 3.95) or treatment as usual (TAU; 8 males, M_age_=77.13 ± 6.38), performing cognitive and physical activities at home for six weeks. The ABILITY intervention additionally included a digital platform enabling communication between the hospital and the patient’s home. Efficiency, such as adherence, perceived fit of demands and skills, usability, and effectiveness measures, including neuropsychological level, neuropsychiatric symptoms, and autonomy in daily living, were collected before (T0), after the treatment (T1), and at the 1-year-follow-up (T2).

**Results:**

The ABILITY program was efficient, with a higher adherence (81% vs. 62%), a higher perceived fit of demands and skills than TAU (*p*<.05), and a good level of technology usability. In terms of effectiveness, a treatment effect (ABILITY > TAU) emerged on the global cognitive level, especially in language, executive functions, and memory domains. Moreover, a treatment carry-over effect (1-year follow-up) was observed in global cognitive functions (especially language) (ABILITY > TAU), behavioral symptoms, and caregiver distress (TAU > ABILITY).

**Conclusions:**

Our preliminary findings suggest that ABILITY is a promising eHealth intervention to improve at-home treatment adherence and to preserve cognitive and behavioral abilities.

## Key Messages

- People in the Alzheimer’s Disease continuum facing chronic cognitive disabilities represent an emergency for the healthcare system given the substantial need for long-term rehabilitation;
- This study evaluates a new model of rehabilitation in the continuity of care for people with cognitive disabilities, adopting an asynchronous approach;
- The asynchronous telerehabilitation model may be considered a new frontier for continuity of care, capable of answering the unmet need of scaling up rehabilitation services to the broad population.

## Introduction

Dementia is currently a public health priority at the center of the global action plan (2017–2025), requiring new solutions to support patients and families in managing disabilities related to the disease [1]. Nowadays, the effect of commercially available symptomatic drugs remains controversial. Only a single molecule, aducanumab, has been approved by the FDA as a disease-modifying drug for Alzheimer’s disease (AD), capable of reducing the amyloid plaque burden [2], but its impact on cognitive decline is more modest. In this context, the need to promptly intervene with neurorehabilitation on residual capabilities is well known. Importantly, different non-pharmacological treatments are available for specific levels of cognitive impairments within the AD continuum, including cognitive stimulation [3], multi-stimulation therapies (e.g. [4]), virtual reality rehabilitation (e.g. [5]), group-therapies (e.g. [6]), and art-based interventions (e.g. [7]). However, rehabilitation currently remains a very specialized service for the limited number of patients with access to an institutional (face-to-face or group-oriented) setting. A recent Global Burden Disease study [8] reported the substantial need for treatments in the general population, approximately 1 in 3 people. In response to this unmet need, telehealth solutions are ideal for triggering the migration of care from clinics to patients’ homes and, consequently, scaling up cognitive rehabilitation from a limited number of patients to broader targets. In fact, through mobile devices and algorithms [9], rehabilitation is now agile in bypassing typical barriers obstructing accessibility, quality, and outcomes of care and sustaining patient empowerment in healthcare management outside clinical institutional recovery [10]. This change has important implications in the current COVID-19 pandemic scenario, giving the possibility to provide care despite overburdened healthcare systems and social distancing requirements [11,12]. Acting on the real-life scenario (@home) and creating a community network supporting persons with dementia in their daily lives, these telerehabilitation systems are promising approaches that can affect the patient’s functionality, the caregiver’s quality of life, and the well-being of the carer-caregiver dyad.

The most sustainable telerehabilitation model to scale up rehabilitation is the asynchronous approach that overcomes the need for constant face-to-face interaction. This way, by utilizing a digital platform allowing a double loop communication between the clinic and the patient’s home, the assessment, monitoring, and feedback is guaranteed, and the rehabilitation program is personalized over time, adapting tasks in a patient-tailored manner [13,14]. In particular, the daily outcome can be recorded in the platform’s server, helping the therapist supervise the patient and interact with the delivery system during the entire rehabilitative program, monitoring the training progression. Moreover, the implementation of digital contents for cognitive activities promotes engagement [11] and facilitates measurement of the patient’s performance and progress in terms of accuracy, reaction time, number of repetitions, and time spent in rehabilitative sessions.

Asynchronous models of telerehabilitation require a complex technological ecosystem underlying clinic-home communication, besides the design and development of digital contents, and to date, only a few examples have been validated in the literature [5,14]. Given the novelty of these asynchronous models of telerehabilitation, randomized controlled trials demonstrating the effectiveness of these solutions are still scarce. The present pilot study aimed to explore the efficiency and effectiveness of an innovative technology-enhanced telerehabilitation for people within the AD continuum at mild to moderate stages of cognitive impairment [15]. We investigated the device’s usability, adherence to the rehabilitation program, and safety (efficiency measures). Also, changes in autonomy in daily living, neuropsychological level, and behavioural symptoms (efficacy measures) were compared to treatment as usual (TAU).

## Materials and methods

### Study design

A randomized controlled trial was designed [15] and registered (NCT02746484) according to CONSORT Criteria (see the CONSORT Checklist in Supplementary Material).

The sample size was computed according to previous multicenter controlled studies [4,16], under the assumption of normal distribution of the outcome scores, considering an α level of .05, a sample size of 30 subjects resulted in a power greater than 70% and therefore judged as adequate for this trial.

The participants assessed as eligible for the study were consecutively enrolled and then randomly allocated to one of the two interventions (ratio 1:1): the ABILITY approach (experimental group) or the TAU (active comparator group).

**Figure 1.**
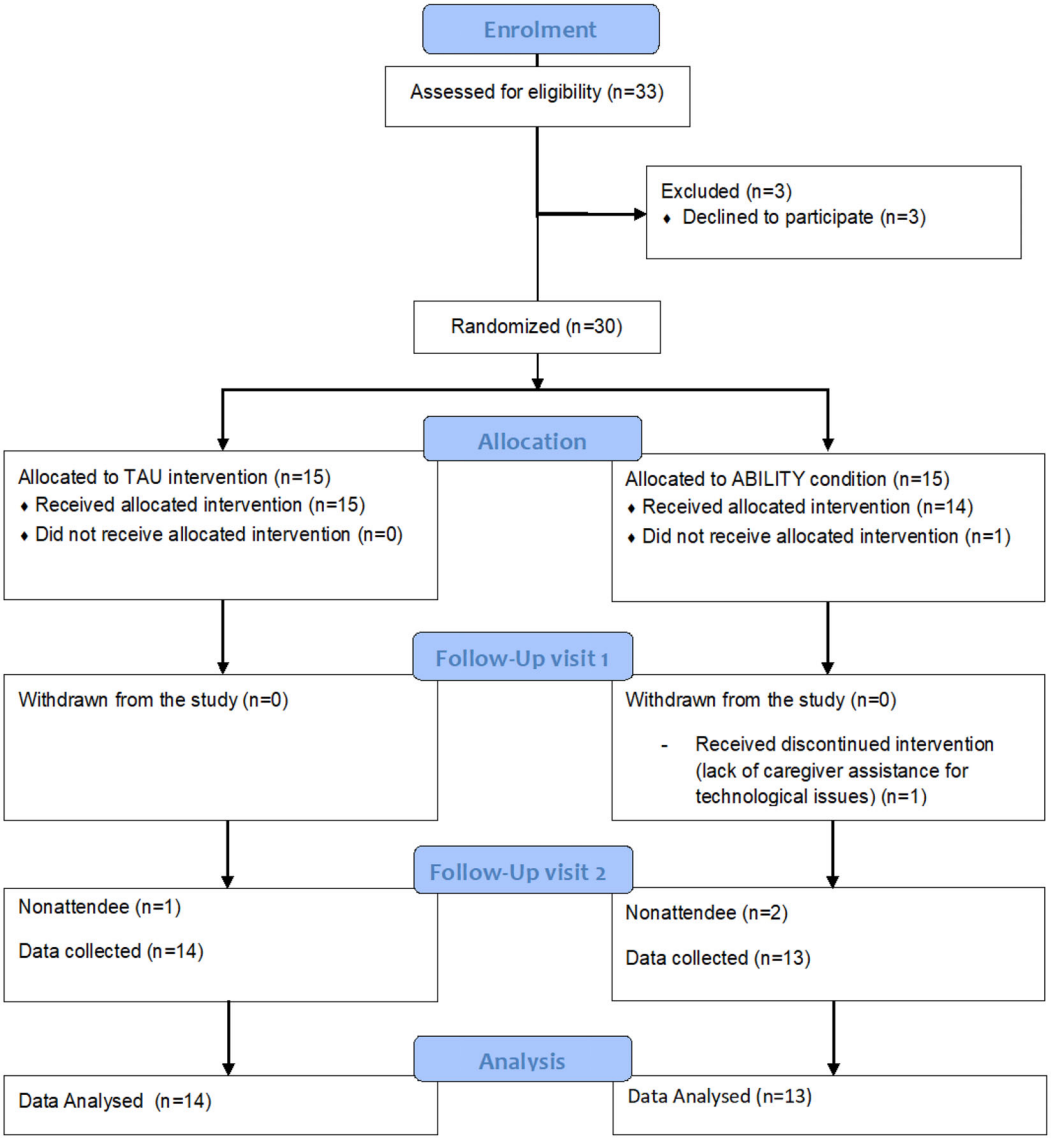
CONSORT diagram of the study.

An independent operator conducted randomization, neither involved in the assessment nor treatment using a computer algorithm (http://www.graphpad.com/quickcalcs/randMenu/). Both groups were evaluated for the measurement of primary and secondary outcome measures at baseline (T0), after rehabilitation (eight weeks from baseline, T1), and after the follow-up period (12 months from baseline, T2) (Figure 1).

Since participants could not be blinded to their treatment allocation, they were instructed not to discuss the nature of their intervention with the researchers in charge of the assessments. Outcome measures were collected by a neuropsychologist blinded to group allocation.

### Characteristics of participants

Thirty-three people with Mild Cognitive Impairment (MCI) or mild dementia within the AD continuum were consecutively enrolled in the study by neurologists during periodical medical screening at the memory clinic at IRCCS Don Gnocchi Foundation center. Inclusion criteria were: diagnosis of mild AD or MCI due to AD [17,18]; Mini-Mental State Examination score > 18 [19]; school attendance ≥ 3 years; availability to participate in a rehabilitative clinical trial; age > 65 years. Exclusion criteria were: dysmetria, visual acuity deficit, auditory perception deficit, and communication deficit that might affect the performance of evaluation tests and rehabilitation activities performance, and stable pharmacological treatment for at least the past three months.

Also, in both groups, the caregiver’s presence (if available) to support the patients during the rehabilitation sessions was welcomed.

The study was approved by IRCCS Don Carlo Gnocchi Foundation Ethics Committee, and all participants of the study read and signed the informed consent sheet to take part in the research.

### Interventions

In both conditions, ABILITY and TAU, rehabilitation adopted a multidimensional approach to promote well-being, cognition, motor, and functional skills in a social environment. In detail, both cognitive and motor activities were included in the rehabilitation program. The cognitive activities were focused on several cognitive domains, such as attention, reasoning, procedural, semantic, and autobiographical memory, executive functions, and visual-spatial skills. The motor activities included motor exercises adapted to be executed at home and aerobic activities to be carried out outside the home to train physical capabilities (for further details, see the study protocol [15]). Subjects were instructed to perform cognitive activities five days per week (for about 20–30 min per day) and motor exercises three days per week (for about 15–25 min per day). Before starting the six weeks of treatment, participants and caregivers allocated in both ABILITY and TAU groups were invited to the clinic to meet the clinicians and receive instructions concerning the activities, a demonstration of digital device use (for the ABILITY group), and suggestions of strategies to manage motivation decline over time.

**Figure 2.**
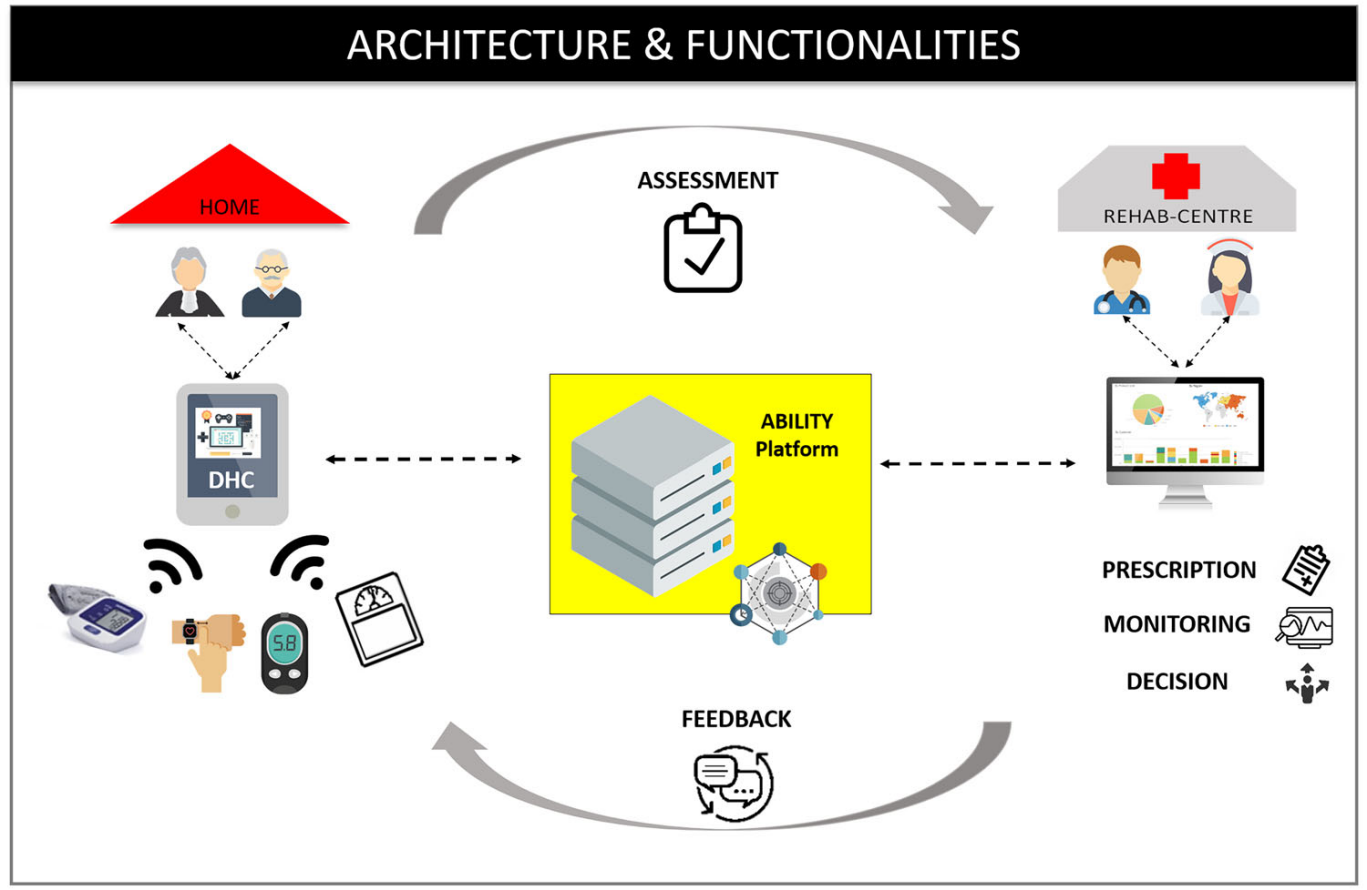
The ABILITY telerehabilitation system: architecture and functionality. DHC: Digital Health Contents for rehabilitation.

The main differences between the ABILITY and the TAU intervention consisted of the tools included in the delivery modality of the rehabilitation at home. The TAU intervention was carried out in a standard manner, and participants received paper and pencil activities for cognitive exercises and written instruction for motor activities. Instead, in the ABILITY group, the intervention was delivered through a digital telerehabilitation platform (see Figure 2). By accessing the platform, the patient found the prescribed rehabilitation activities to be performed each day. The therapist was able to monitor cognitive activity results and vital parameters and program and manage the rehabilitation program of the patient. The digital platform was certified medical equipment, the ABILITY Telerehabilitation Platform (https://abmedica.it/prodotti-ab-medica/maia), to ensure both safety and privacy. The architecture and functionality of the ABILITY platform are illustrated in Figure 2. The platform architecture can be conceptually divided into three layers: 1) Patient side: data to assess the rehabilitation progress are recorded and stored in the ABILITY technologies; 2) Middleware layer: data from heterogeneous devices and systems converge into the rehabilitation platform; 3) Clinician side: data converged into the platform are utilized to monitoring and providing feedback to the patient and personalizing rehabilitation by the clinicians.

The TAU and the ABILITY treatment were different also for the management of activities’ difficulty level: the ABILITY rehabilitation presented an adaptive incremental difficulty level of cognitive activities based on the subject’s performance plus the subject’s reported perceived difficulties (ABILITY); the TAU provided a fixed incremental difficulty level over time independently from the subject’s performance and perspective. In detail, for both ABILITY and TAU treatment, each activity was structured into five levels of difficulty. For the ABILITY cognitive activity, a specific criterium to advance to the next level was set by an algorithm considering both i) the subject’s performances (i.e. the number of errors, the time spent to perform the activity) and ii) the level of perceived difficulties reported by the patient at the end of each activity. For the TAU rehabilitation, the level of difficulty incremented each week in a standard modality.

Another difference between the ABILITY and TAU was the instructions for the physical activities, which consisted of video tutorials for the ABILITY, versus written instructions for the TAU.

Finally, only the ABILITY treatment provided telemonitoring of vital parameters (body weight, oxygen blood level, blood pressure, and heart rate) by biometric devices (a sphygmomanometer for blood pressure measurement, a pulse oximeter for recording oxygen blood level and heart rate, a scale for measurement of body weight to be used once a day, and a wearable device to track the physical and sleep activity for the entire period of the trial), integrated into the ABILITY platform to be constantly monitored from the clinic. Once a week, an alert on the tablet was set to remind the ABILITY group to measure the vital parameters. The TAU intervention included a paper-and-pencil diary to be filled by the subject with data on vital parameters.

Only the ABILITY group was instructed to wear an activity tracker 24/24h for the whole treatment period, except for 2 h per week (time to recharge the device).

### Measurements

Patients and their caregivers were assessed at baseline (T0), after eight weeks of treatment (T1), and after 12 months after the baseline (T2) by a neuropsychologist blinded to the patient’s assignment to groups (ABILITY vs. TAU). Both patients and caregivers were asked about the usability of the ABILITY technology for home settings. Patients were evaluated individually with a neuropsychological battery, autonomy in daily living, and behavioral symptoms, as detailed below.

Efficiency Output Measures: the System Usability Scale (SUS; [20–22]) was administered to caregivers and patients who underwent ABILITY rehabilitation to test the technology’s ease of learning and use. Treatment adherence was assessed by calculating the percentage of sessions attended within the timeline of 6 weeks, five times per week, for each subject. The balance between abilities required during the rehabilitation activities and the patient’s perceived ability to perform them was registered by administering a 6-points scale item (1 = too easy to perform, 6 = too difficult to perform). A score of 3 was considered a perfect balance between required and own abilities, while a score of < or > 3 was an unbalanced level of difficulty in rehabilitation activities. Finally, adverse events during the trial were registered by interviewing patients and caregivers in a planned weekly phone call to evaluate the treatment safety.

Patients Neuropsychological Assessment: the Montreal Cognitive Assessment (MoCA [23]) test was administered to measure the global cognitive level. The Verbal fluency test, phonemic and semantic fluency (FAS [24], CAT[25]), was used to test language ability. The frontal executive functions were assessed through the Trail Making Test (TMT parts A and B [26]). Finally, the Free and Cued Selective Reminding Test (FCSRT [27]) was administered to assess the immediate free recall (IFR), the immediate total recall (ITR), the delayed-free recall (DFR), and the delayed total recall (DTR) (see also Table 1).

**Table 1.** Output and outcome measurements.

Domain	Measure	Scoring	Score range	and cut-off
Global Cognitive Level	MoCA	Total score	0–30	17.363
Language	FAS	Total score	0–∞	17.35
	CAT	Total score	0–∞	24.00
Executive Functions	TMT	PART A time	0–∞	93.00
		PART B time	0–∞	282.00
Memory	FCSRT	IFR	0–36	19.59
		ITR	0–36	34.00
		DFR	0–12	6.31
		DTR	0–12	10.00

MoCA: Montreal Cognitive Assessment [23]; FAS: letter verbal fluencies [24]; CAT: categorical verbal fluencies [25]; TMT: Trail Making Test [26]; FCSRT: Free and Cued Selective Reminding Test [27]; IFR: Immediate Free Recall; ITR: Immediate Total Recall; DFR: Delayed Free Recall; DTR: Delayed Total Recall.

Autonomy in daily living and behavioral symptoms: the level of autonomy in daily living was evaluated through the Activities of Daily Living Inventory (ADCS/ADL [28,29]) by interviewing caregivers. The severity and frequency of behavioral symptoms were registered *via* the Neuropsychiatric Inventory (NPI [30]) by asking the caregiver to report symptoms of dementia.

### Statistical analysis

The Shapiro-Wilk test was utilized to assess the distribution of variables, and accordingly, parametric or non-parametric analyses were conducted as appropriate. Summary statistics were utilized to describe the demographic characteristics of the two groups (ABILITY and TAU). Also, independent t-test and Chi-squared analyses were run to ensure that the two groups were comparable at baseline evaluation in terms of age, level of education, neuropsychological profile, and sex distribution. Summary statistics, frequencies, and paired and unpaired comparisons (independent t-test or Mann-Whitney) were reported to test the efficiency of ABILITY versus TAU.

To test the effectiveness of the ABILITY treatment on primary and secondary neuropsychological outcome measures (see NCT02746484), we computed delta changes and obtained period effect (PE), treatment effect (TE), follow-up effect (FUE), and carry-over effect (COE). T0 was the baseline evaluation; T1 was the post-treatment evaluation (after 6 weeks), and T2 was the follow-up evaluation (1 year after baseline). PE was calculated on the whole group (ABILITY + TAU group) to measure outcome changes along with time points, both during the treatment period (PE_T1-T0_) and follow-up period (PE_T2-T1_). TE was calculated to measure each group’s change in outcomes during the treatment period separately (TE_ABILITY_; TE_TAU_). The Follow-up effect (FUE) was also derived by computing delta changes between T2 and T1 separately for groups (FUE_ABILITY_; FUE_TAU_). COE reflected the outcome change from baseline to the follow-up period separately per group by summing TE to FUE. We ran paired sample comparison (paired t-test or Wilcoxon) to test the differences between PE_T1-T0_ and PE_T2-T1_. Unpaired comparison (independent t-test or Mann-Whitney) was performed to test group differences in TE and COE. The effect size was reported for statistically significant findings (Cohen’s *d* was reported for parametrical comparison tests, while the Rank biserial correlation was reported for non-parametrical comparison). Given the small sample size and the small proportion of missing data (1%), no multiple imputation approach was adopted to handle missing data.

## Results

### Participants

Three of the 33 participants recruited for the study declined to start the treatment at home. In total, 30 subjects along the neurobiological AD continuum, in particular, MCI (*n* = 11) or mild AD (*n* = 19) within the AD continuum, took part in the study. The ABILITY group (*n* = 15) comprised 9 people with MCI and 6 people with AD (χ^2^ = 0.267, *p* = 0.606). TAU group (*n* = 15) included 10 people with MCI and 5 people with AD (χ^2^ = 1.067, *p* = 0.258). These two groups were balanced for sex distribution, age, education, and global cognitive level. Table 2 reports details of the groups’ characteristics at baseline.

**Table 2.** Demographic characteristics of groups at baseline and comparison results.

	ABILITY	TAU	test	*p* Value
*Demographics*				
N	15	15	-	-
Males (%)	6 (40.0%)	8 (53.3%)	0.134°	0.714
Age (M ± SD)	78.2 ± 3.95	77.13 ± 6.38	0.551^^^	0.586
Education(M ± SD)	9.33 ± 4.01	7.60 ± 2.67	1.393^^^	0.174
*Global Cognitive Level*				
MMSE(M ± SD)	25.24 ± 3.17	24.93 ± 3.86	0.234^^^	0.817
MoCA(M ± SD)	18.24 ± 4.40	17.96 ± 5.76	0.151^^^	0.881
*Language*				
CAT (M ± SD)	22.78 ± 8.92	24.63 ± 9.65	0.545^^^	0.590
FAS (M ± SD)	24.05 ± 6.66	26.79 ± 8.31	0.994^^^	0.329
*Executive Functions*				
TMTA (M ± SD)	93.80 ± 135.43	119.27 ± 184.13	118.50^§^	0.806
TMTB (M ± SD)	219.40 ± 165.46	270.93 ± 199.69	94.00^§^	0.461
*Memory*				
IFR (M ± SD)	15.51 ± 7.61	16.45 ± 9.21	0.305^^^	0.763
ITR (M ± SD)	31.00 ± 5.72	31.00 ± 7.06	83.50^§^	0.233
DFR (M ± SD)	4.21 ± 3.42	5.18 ± 4.17	0.752^^^	0.458
DTR (M ± SD)	10.00 ± 2.65	9.80 ± 3.08	105.00^§^	0.775

TAU: treatment as usual; M: mean; N: number; SD: standard deviations; **°**: χ^2^ chi-squared; **^**: t independent t-test; ^§^: U Mann-Whitney test. CAT: categorical verbal fluencies; DFR: Delayed Free Recall; DTR: Delayed Total Recall; FAS: letter verbal fluencies; IFR: Immediate Free Recall; ITR: Immediate Total Recall; MoCA: Montreal Cognitive Assessment; PE: Period Effect; TAU: Treatment as usual; TMTA: Trail Making Test – part A; TMTB: Trail Making Test – part B.

### Efficiency output measures

In terms of adherence, the two groups presented different trends. Specifically, we registered a difference between groups in the total number of sessions completed within the timeline of the six weeks of treatments (*U* = 61.00, *p* = 0.034, two-tailed, rank biserial correlation = 0.46). The ABILITY group reported a mean adherence of 0.81 ± 0.32 versus a mean adherence of 0.62 ± 0.28 in the TAU group. By focusing on the degree of adherence for each week of treatment, we observed a significant group difference in the fifth (ABILITY > TAU: *U* = 57.00, *p* = 0.01, two-tailed, rank biserial correlation= 0.49) and the sixth (ABILITY > TAU: *t* = 3.82, two-tailed, *p* < 0.001, Cohen’s *d* = 1.42) week.

Regarding the perceived difficulty level of activities for individual capabilities (balancing), we found a significant difference between groups. By comparing the balancing of activities for each week of treatment, we observed significant group differences in the fourth (TAU > ABILITY: *t* = 2.12, *p* = 0.046, Cohen’s *d* = 0.89) and the fifth (TAU > ABILITY: *t* = 2.20, *p* = 0.041, Cohen’s *d* = 0.96) week. Table 3 shows each group’s mean adherence and perceived balancing over the six weeks of treatment and a comparison between groups.

**Table 3.** Adherence and balancing in the two groups per week of treatment.

	Week 1	Week 2	Week 3	Week 4	Week 5	Week 6
*Adherence*						
ABILITY (M ± Sd)	0.91 ± 0.26	0.84 ± 0.34	0.80 ± 0.38	0.76 ± 0.40	0.77 ± 0.40	0.81 ± 0.33
TAU (M ± SD)	0.93 ± 0.12	0.72 ± 0.37	0.72 ± 0.38	0.65 ± 0.41	0.41 ± 0.38	0.29 ± 0.40
Comparison	1.106	1.52	1.029	1.132	3.075	3.824
*p*	0.632	0.178	0.289	0.237	**0.008**	**<0.001**
*Perceived balancing*						
ABILITY (M ± SD)	2.80 ± 0.39	2.87 ± 0.32	2.93 ± 0.45	2.89 ± 0.45	3.05 ± 0.46	3.32 ± 0.67
TAU (M ± SD)	2.92 ± 0.44	3.10 ± 0.42	3.36 ± 0.59	3.36 ± 0.60	3.63 ± 0.73	3.40 ± 0.73
Comparison	0.70	1.63	2.02	2.12	2.198	0.24
*p*	0.491**^^^**	0.278^§^	0.054^^^	**0.046^^^**	**0.041^^^**	0.842^§^

M: mean; SD: standard deviation; ^: t independent t-test; §: U Mann-Whitney test. *p*-value < 0.05 are reported in bold.

Regarding the perceived usability of technology, the adjective rating of the SUS scale was considered according to Bargor et al. [20]. The results, reported in Table 4, showed an okay-to-excellent level of usability in patients (33% of patients judged the system ‘Okay’, 47% ‘Good’, 20% ‘Excellent’) and a good-to-excellent level of usability in caregivers (14.28% of caregivers judged the system ‘Okay’, 35.71% ‘Good’, 28.57% ‘Excellent’, 21.44 ‘Best imaginable’). Concerning Usability and Learnability subscores, results revealed high usability both for patients (*M* = 2.39, sd = 0.46) and caregivers (*M* = 2.71, sd = 0.72), while only caregivers reported a high score of learnability (caregivers: *M* = 3.43, sd = 0.94; patients: *M* = 1.80, sd = 1.13).

**Table 4.** System usability scale results.

		L1	L2	L3	L4	L5	L6	L7
SUS	Patients	0%	0%	0%	33%	47%	20%	0%
	Caregivers	0%	0%	0%	14%	36%	29%	21%

L1: worst imaginable; L2: awful; L3: poor; L4: ok; L5: good; L6: excellent; L7: best imaginable; SUS: System Usability Scale.

Regarding the safety of the ABILITY approach, no adverse events were registered during the trial.

### Outcome measures

#### Neuropsychological level

Table 5 shows PE results on the neuropsychological functions. The paired-sample comparison revealed a statistically significant difference in delta changes between treatment and follow-up period in memory outcomes. Delta changes demonstrated a major increment during treatment compared to follow-up period in IFR (*p* < 0.001, Cohen’s *d* = 0.686), ITR (*p* = 0.017, Rank biserial correlation = 0.522) and DTR (*p* = 0.014, Cohen’s *d* = 0.456).

**Table 5.** Period effect results on neuropsychological level.

	*PE_T1-T0 _* M-change	*PE_T2-T1 _* M-change	Follow-up > Treatment *p*-value	Follow-up < Treatment *p*-value
*Global Cognitive Level*				
MoCA	−0.12	0.15	0.517	0.483
*Language*				
FAS	0.50	−0.31	0.860	0.146
CAT	−0.42	−0.15	0.446	0.554
*Executive Functions*				
TMT-A	4.46	23.73	0.137	0.661
TMT-B	3.77	−2.54	0.595	0.086
*Memory*				
IFR	1.88	−2.42	0.999	**<0.001**
ITR	0.15	−3.61	0.988	**0.020**
DFR	0.05	−0.17	0.633	0.367
DTR	0.00	−1.00	0.986	**0.014**

CAT: categorical verbal fluencies; DFR: Delayed Free Recall; DTR: Delayed Total Recall; FAS: letter verbal fluencies; IFR: Immediate Free Recall; ITR: Immediate Total Recall; MoCA: Montreal Cognitive Assessment; PE = Period Effect; TAU: Treatment as usual; TMT-A: Trail Making Test – part A; TMT-B: Trail Making Test – part B. Significant *p* values are reported in bold.

Focusing on group differences (ABILITY vs TAU) in treatment effects (see Table 5), results of unpaired sample analysis highlighted higher treatment period delta changes (T1-T0) in ABILITY than TAU group in MoCA (ABILITY > TAU: *p* = 0.022, Cohen’s *d* = 0.784), CAT (ABILITY > TAU: *p* = 0.044, Cohen’s *d* = 0.658), ITR (ABILITY > TAU: *p* = 0.020, Rank biserial correlation = 0.448) and TMT-A (ABILITY > TAU: *p* = 0.016, Cohen’s *d* = 0.838).

Considering group differences in COE effect, we found a higher delta change in ABILITY than TAU group in MoCA (ABILITY > TAU: *p* = 0.021, Cohen’s *d* = 0.845) and CAT (ABILITY > TAU: *p* = 0.036, Cohen’s *d* = 0.737) (see Table 6).

**Table 6.** TE and COE results.

	*TE*				*COE*			
	Delta M-change		ABILITY > TAU *p*-value	TAU > ABILITY *p*-value	Delta M-change		ABILITY > TAU *p*-value	TAU > ABILITY *p*-value
	ABILITY	TAU			ABILITY	TAU		
*Global Cognitive Level*								
MoCA	0.53	−1.14	**0.022**	0.978	1.31	−1.85	**0.021**	0.979
*Language*								
FAS	0.93	0.43	0.364	0.636	2.00	−1.62	0.093	0.907
CAT	1.33	−2.36	**0.044**	0.956	1.38	−2.54	**0.036**	0.964
*Executive Functions*								
TMT-A	−4.60	13.29	0.984	**0.016**	−4.85	61.23	0.917	0.091
TMT-B	−6.27	10.43	0.901	0.099	−27.46	29.92	0.866	0.145
*Memory*								
IFR	2.40	1.21	0.209	0.791	0.02	−1.10	0.294	0.706
ITR	0.80	−1.93	**0.020**	0.982	−2.46	−4.46	0.388	0.632
DFR	0.067	−0.23	0.387	0.631	−0.23	−0.01	0.601	0.399
DTR	0.00	−0.50	0.315	0.701	−0.92	−1.08	0.626	0.394

MoCA: Montreal Cognitive Assessment; FAS: letter verbal fluencies; CAT: categorical verbal fluencies; TMT-A: Trail Making Test – part A; TMT-B: Trail Making Test – part B; IFR: Immediate Free Recall; ITR: Immediate Total Recall; DFR: Delayed Free Recall; DTR: Delayed Total Recall; TAU: Treatment as usual; TE: Treatment Effect; COE: Carry-Over Effect. Significant *p* values are reported in bold.

#### Daily living autonomy and behavioral symptoms

In behavioral functioning, paired comparison showed a major increment during treatment compared to the follow-up period in ADCS total score (*p* = 0.018, Cohen’s *d* = 0.479) in the whole group, and specifically, in both communication (*p* = 0.034, Cohen’s *d* = 0.410) and outdoor activities (*p* = 0.022, Cohen’s *d* = 0.459) scores (see Table 7).

**Table 7.** PE results on autonomy in daily living and behavioral symptoms.

	PE_T1-T0 _ M-change	PE_T2-T1_ M-change	Follow-up > Treatment *p*-value	Follow-up < Treatment *p*-value
*Autonomy*				
ADCS Total	−0.58	−8.23	0.982	**0.018**
Basic activities	−0.25	−1.18	0.901	0.099
Household activities	−0.42	−3.32	0.950	0.050
Communication	0.63	−1.77	0.966	**0.034**
Outside activities	−0.54	−1.95	0.978	**0.022**
*Behavioral symptoms*				
NPI				
Symptoms	3.27	−3.46	0.772	0.237
Caregiver distress	0.23	−0.36	0.587	0.413

ADCS: Activities of Daily Living Inventory; NPI: Neuropsychiatric Inventory; PE: Period Effect. Significant *p* values are reported in bold.

Regarding TE on behavioral functions, unpaired analysis revealed a significant group difference in COE in NPI, in terms of both frequency of symptoms (TAU > ABILITY: *p* = 0.022, *Rank biserial correlation* = 0.517) and caregiver distress (TAU > ABILITY: *p* = 0.036, Cohen’s *d* = 0.812) (Table 8).

**Table 8.** TE and COE results on autonomy in daily living and behavioral symptoms.

	TE				COE			
	M-change		ABILITY > TAU *p*-value	TAU > ABILITY *p*-value	M-change		ABILITY > TAU *p*-value	TAU > ABILITY *p*-value
	ABILITY	TAU			ABILITY	TAU		
*Autonomy*								
ADCS Total	−1.64	0.90	0.732	0.288	−2.83	−14.00	0.765	0.235
Basic activities	−0.43	0.00	0.863	0.154	−0.83	−2.20	0.863	0.154
Household activities	−1.14	0.60	0.936	0.072	−1.83	−5.10	0.936	0.823
Communication	0.714	0.50	0.428	0.572	0.83	−3.20	0.428	0.921
Outside activities	−0.79	−0.20	0.798	0.221	−1.00	−3.50	0.718	0.961
*Behavioral symptoms*								
NPI								
Symptoms	1.07	6.20	0.750	0.269	−4.50	5.00	0.750	**0.022**
Caregiver distress	0.57	0.50	0.486	0.514	−2.92	3.20	0.486	**0.036**

ADCS: Activities of Daily Living Inventory; NPI: Neuropsychiatric Inventory; TE: Treatment Effect; COE: Carry-Over Effect. Significant *p* values are reported in bold.

## Discussion

In the present study, we explored the efficiency and effectiveness of a telerehabilitation model with an asynchronous modality, named ABILITY, to deliver rehabilitation care in a well-characterized cohort of subjects with mild to moderate stages of the AD continuum. We found that the ABILITY telerehabilitation treatment efficiently guarantees patients’ adherence to the treatment, an adaptable level of difficulty, and a usable and safe experience.

Concerning adherence, the ABILITY group fully followed six weeks of intervention at home, while the active control group trended towards disengagement after the fourth week. Also, the adaptability of the cognitive activities of the ABILITY program guaranteed a perception of balance between perceived own and the required ability to perform planned sessions. On the contrary, we registered a perceived unbalanced level of difficulty in the paper-and-pencil activities during the last sessions in the control group. These results demonstrate the beneficial effect of delivering telerehabilitation both for the engagement of patients and for the potential management of the rehabilitation activity over time, in accordance with the literature [31,32]. Tailoring cognitive activities to the patient’s capability during the rehabilitation program is essential to guarantee adherence to the treatment and patient engagement, as suggested by WHO recommendations [33].

The ABILITY system was judged as usable by caregivers and patients, although learnability was lower for the latter, indicating a higher amount of time to learn how to use the product. This is not unexpected since the level of cognitive impairment has been demonstrated to be inversely related to everyday technology usage [29,34]. However, the imminent intergenerational phenomenon leads us to argue that patients will have a solid familiarity with digital technology in everyday life in future decades. Given the potential of applying digital health platforms in telerehabilitation [14,35,36] for people with dementia [32,37], future studies should consider adopting alternative research approaches to ensure an adequate user experience with technology. Accordingly, user-centered designs [38,39] are a widely accepted approach for implementing technological rehabilitation solutions, including different Design-Evaluation-Redesign cycles that iteratively involve end-users and healthcare and technology professionals in the creation and refinement of technical systems [40].

Moreover, safety, an essential efficiency component, was confirmed because no adverse events were registered during the trial. Our effectiveness pilot findings suggested the ABILITY treatment effect at multidimensional levels, both in the short and long term. In fact, our data revealed a major impact of the treatment at T1 in the ABILITY group compared to the TAU group, not only at the global cognitive level but also in language, motor perceptual, and neurocognitive memory domains. Interestingly, we observed that only the ABILITY group maintained these latter effects over the follow-up period, suggesting the long-term capacity of the benefits provided by the treatment, especially in the global cognitive level and language domain. On the contrary, although following the conventional recommendations for continuity of care, the control group worsened cognitive functioning over time. These 1-year follow-up results align with a previous study [5] that showed a long-term effect of telerehabilitation after 3-months of follow-up with respect to maintenance of cognitive functions. Moreover, our results showed a more prolonged effect (12 months from enrollment). This finding is also in line with a recent meta-analysis [41] on the effectiveness of cognitive training on the cognition of people with mild to moderate dementia. In our study, the benefit of medium-term rehabilitation was mainly found in global cognitive level and verbal fluencies. Nevertheless, this contribution underlined only slight to moderate positive effects of cognitive treatments on cognition, whereas our findings highlighted large effect sizes (Cohen’s *d* > 0.74) related to the carry-over treatment effect on MoCA and categorical fluencies. Additionally, the ABILITY approach is based on multidomain activities, specific cognitive rehabilitation performed three days per week, and motor activities two days per week. As previous studies have shown the relationship between motor and cognitive functions [35,42], it is plausible to assume that motor enhancement impacted the cognitive domain in ABILITY telerehabilitation.

The maintenance of the cognitive level for one year was also reflected by behavioral symptoms, whose frequency differed between groups after one year, with a considerable worsening in the control group, as reported by the caregivers. Consistent effects of ABILITY rehabilitation were also observed for caregivers, who reported decreased perception of distress related to the carer’s assistance, in line with a recent study investigating frailty in older adults [42]. This is a considerable finding, given that the distress related to the carer’s assistance is tightly linked with experiences of anxiety and depression [43–47]. Validating treatments that provide beneficial effects to the dyad also allows for the preservation of the integration of the patients in their social community [48–50]. This can also positively influence the health care system and society at large [50].

These findings are relevant in that they likely suggest, for the first time with a 1-year trial, the potential of telerehabilitation with an asynchronous approach capable of scaling up rehabilitation to a broader population of patients outside the clinic. In fact, ABILITY telerehabilitation enables remote monitoring, feedback, and the modification of individualized patient-centered rehabilitation over time according to the patient’s actual progress.

Our study is limited to pilot evidence, and future studies need to confirm the results with a broader sample of people with a more varied degree of disability. Also, future telerehabilitation trials should include a phase of familiarization with technologies for the participants before starting the rehabilitation program. This would minimize the potential lack of familiarity with technology in older people, thus enhancing human-technology interaction. Moreover, an additional comparison between usual care clinic treatment and home-based personalized programs will be helpful for future analyses of costs and access. Finally, further contributions will also address the subjective experience of patients and caregivers during home-based personalized programs adopting a mixed (qualitative-quantitative) model approach.

The results from this pilot randomized controlled study suggest ABILITY telerehabilitation is efficient and likely effective. The proposed treatment is a promising intervention for enhancing environmental, social, and functional resources and preserving cognitive abilities.

## Supplementary Material

Supplemental Material

## Data Availability

Data can be obtained upon reasonable request to the corresponding author.
